# Retrospective Evaluation of Bayesian Risk Models of LVAD Mortality at a Single Implant Center

**DOI:** 10.3389/fmed.2018.00277

**Published:** 2018-10-02

**Authors:** Lisa C. Lohmueller, Manreet K. Kanwar, Stephen Bailey, Srinivas Murali, James F. Antaki

**Affiliations:** ^1^Computer Science, Carnegie Mellon University, Pittsburgh, PA, United States; ^2^Cardiovascular Institute, Allegheny General Hospital, Pittsburgh, PA, United States; ^3^Biomedical Engineering, Cornell University, Ithaca, NY, United States

**Keywords:** left ventricular assist device, Bayesian, mortality prediction, patient selection, heart failure, INTERMACS

## Abstract

Use of a left ventricular assist device (LVAD) can benefit patients with end stage heart failure, but only with careful patient selection. In this study, previously derived Bayesian network models for predicting LVAD patient mortality at 1, 3, and 12 months post-implant were evaluated on retrospective data from a single implant center. The models performed well at all three time points, with a receiver operating characteristic area under the curve (ROC AUC) of 78, 76, and 75%, respectively. This evaluation of model performance verifies the utility of these models in “real life” scenarios at an individual institution.

## Introduction

Heart failure is a chronic, progressive condition that affects over 6 million Americans. It is characterized by a decline in function of the heart to pump enough blood to perfuse the body (1). As the condition progresses, treatments may escalate from risk factor modification and oral medications to intravenous inotropes and surgical interventions, such and mechanical heart-assist pumps and heart transplantation (2). Heart transplantation is the gold standard treatment for end stage heart failure; however, donor heart supply is limited and not all patients are eligible for transplant, due to their age, comorbid conditions, or lifestyle choices. As an alternative, advanced heart failure patients may receive a durable left ventricular assist device (LVAD) as a bridge to transplant (BTT) or as a destination therapy (DT) (3).

LVADs can improve quality of life and increase patient survival (4, 5), but also require changes in daily life, a significant investment of time and money, and are associated with risks of adverse events (6). These tradeoffs underscore the importance of careful patient selection, for which predictive models can serve as an important component of risk assessment.

We recently developed models to predict post-LVAD mortality at 1, 3, and 12 months after implant (7) using the data from the Interagency Registry for Mechanically Assisted Circulatory Support (INTERMACS), the largest registry of retrospective LVAD patient data in the United States (4). The models were developed using Bayesian analysis and validated with a subset of registry data that was withheld from the model derivation. While use of the large registry dataset provides a robust model, it obscures institution-dependent differences in patient selection, care, and outcomes. Use of a personalized decision support tool in a “real world” clinical setting is necessary to understand its applicability at individual institutions.

Additionally, the INTERMACS registry has missing data and entry errors. The extent to which missing data affects the performance of the Bayesian predictive models is unknown; therefore, a carefully checked and evaluated dataset from a single clinical site was used to measure model performance.

This study was undertaken to establish the performance of our Bayesian models for LVAD mortality at a single institution with a complete, retrospective patient data set. The goal of this work was to prove the utility of the models for eventual use in prospective patient risk assessment.

## Methods

### Data acquisition and cleaning

We acquired site-specific INTERMACS data for 100 consecutive patients who received a CF-LVAD at Allegheny General Hospital (AGH) between 2014 and 2015. Patients signed consent forms for their data to be collected in INTERMACS at the time of LVAD implant. A data sharing agreement was established between Carnegie Mellon University (CMU) and AGH to assure the security of protected health information in this study. This study was approved by CMU and AGH's review boards for biomedical research (IRBs).

The time-period was selected to include records with at least 1 year of follow up data. The data was organized into three categories: Pre-Implant, Post-Implant, and Events. Missing or illogical data (outside of feasible range or conflicting with other entries) was manually identified and checked by a data coordinator. Data elements that were designated as “unknown” or “missing” were addressed by reviewing all available patient medical records. In cases where the data could not be found, the data field was denoted as “not recorded.” All units for continuous variables were also checked. Once all 100 patients were verified by the coordinator at AGH, the data set was sent to CMU for analysis.

Data cleaning revealed 9% of all pre-implant information (2,704 out of 28,500 possible fields, 2,850 per patient) was missing or out of range in the patient records. After data cleaning, this was reduced to 4% (1,184) fields that were confirmed as not recorded. This cleaned data set was used for the validation analysis.

### Data pre-processing

Pre-implant continuous data were binned into groups, which were determined during the initial model derivation (7) and briefly described, below. Mortality outcomes were determined for each patient using the Event data for each of the three time points: 1, 3, and 12 months post-LVAD.

### Original model derivation and predictive variables

The models used in this analysis were derived using pre-implant patient information from INTERMACS from January 2012 to December 2015, for adults (over 18 years of age) who received their first primary continuous flow LVAD or LVAD and right ventricular assist device (RVAD) in combination (*n* = 10,277). This time frame was chosen to include current generation continuous flow LVADs and contemporary approaches to patient management. Outcomes for mortality were chosen at 1, 3, and 12 months after primary LVAD implant, to capture early outcomes that may impact hospital performance and reimbursement (8) (1 and 3 months) and long-term outcomes (12 months).

Naïve Bayes (NB) models were derived for each time point using a training dataset consisting of 80% of the records selected at random (*n* = 8,222). The remaining 20% (*n* = 2,055) were held aside for model validation. Continuous variables were discretized using either expert binning, equal frequency, or equal width binning to achieve the maximum information gain for each variable with respect to the model time-point. Feature selection was performed using information gain on the training data. Models were learned using the NB method in GeNie 2.2 (BayesFusion, Pittsburgh, PA). Each model was optimized by running 10-fold cross validation and removing variables with low diagnostic value (as measured in GeNie) until the area under the receiver operator characteristics curve (ROC AUC) dropped precipitously. The final NB models had 28, 26, and 21 predictive variables for the 1, 3, and 12-months outcomes, respectively, with 36 total unique variables. The resulting Bayesian models are illustrated in Supplemental Material.

Variables with the highest diagnostic value for 1-month post-LVAD mortality were concomitant RVAD implant, total number of events during the implant hospitalization, platelet count, bilirubin, aspartate aminotransferase, and INTERMACS profile. For the 3-months mortality model, the highest diagnostic value variables were concomitant RVAD implant, age, blood urea nitrogen, hemoglobin and INTERMACS profile. For the long-term mortality prediction, the most associated variables were age, blood urea nitrogen, hemoglobin, device strategy (DT), and concomitant RVAD implant. The diagnostic value for each variable in the model is captured in Supplemental Material.

### Analysis of patient population

The patient population from the AGH study cohort was compared to the LVAD patient population from INTERMACS that was used for original model derivation and validation. Fisher's exact test, Pearson's chi-square and student's *t*-test were used to compare the populations in SPSS (IBM).

### Model validation and comparison

The complete AGH data sets were used to measure the Bayesian mortality model performance for each time point, using test validation in GeNie (BayesFusion, Pittsburgh, PA). The resulting ROC AUCs were compared to the original model validation performance using DeLong's test (9) with the pROC package in R.

## Results

The patient cohort at AGH was similar to the overall INTERMACs population in terms of patient age and gender (Table 1). The main difference between cohorts were the distribution of INTERMACS profiles (*p*-value < 0.001) and the distribution of device strategies (*p*-value < 0.001). The AGH population had a larger proportion of INTERMACS profiles 1 and 2 and a larger proportion of likely bridge to transplant (BTT) patients. The rate of mortality events was similar to the INTERMACs population for all three end-points.

**Table 1:** Comparison of AGH patient cohort with overall INTERMACS registry.

**Characteristic**		**AGH patients (*****n*** = **100)**		**INTERMACS patients (*****n*** = **10,277)**		***p*-value**
		**Mean (SD)**		**Mean (SD)**		
Age		56.2 (12.7)		56.9 (13)		*0.592*
		***n***	**%**	***n***	**%**	
Gender	Male	73	73%	8,044	78%	*0.280*
Ischemic Etiology	Yes	52	52%	4,637	45%	*0.189*
INTERMACS	1	20	20%	1,671	16%	
	2	48	48%	3,548	35%	
	3	14	14%	3,318	32%	
	4–7	18	18%	1,740	17%	*<0.001*
Device Strategy	BTT likely	67	67%	5,261	51%	
	BTT unlikely	5	5%	267	3%	
	DT	25	25%	4,658	45%	
	Other	3	3%	91	1%	* < 0.001*
Mortality	1-month	4	4%	540	5%	*0.820*
	3-months	8	8%	976	9%	*0.733*
	12-months	18	18%	1,849	18%	*1.000*

*INTERMACS, Interagency Registry for Mechanically Assisted Circulatory Support; BTT, bridge to transplant; DT, destination therapy*.

**Figure 1:**
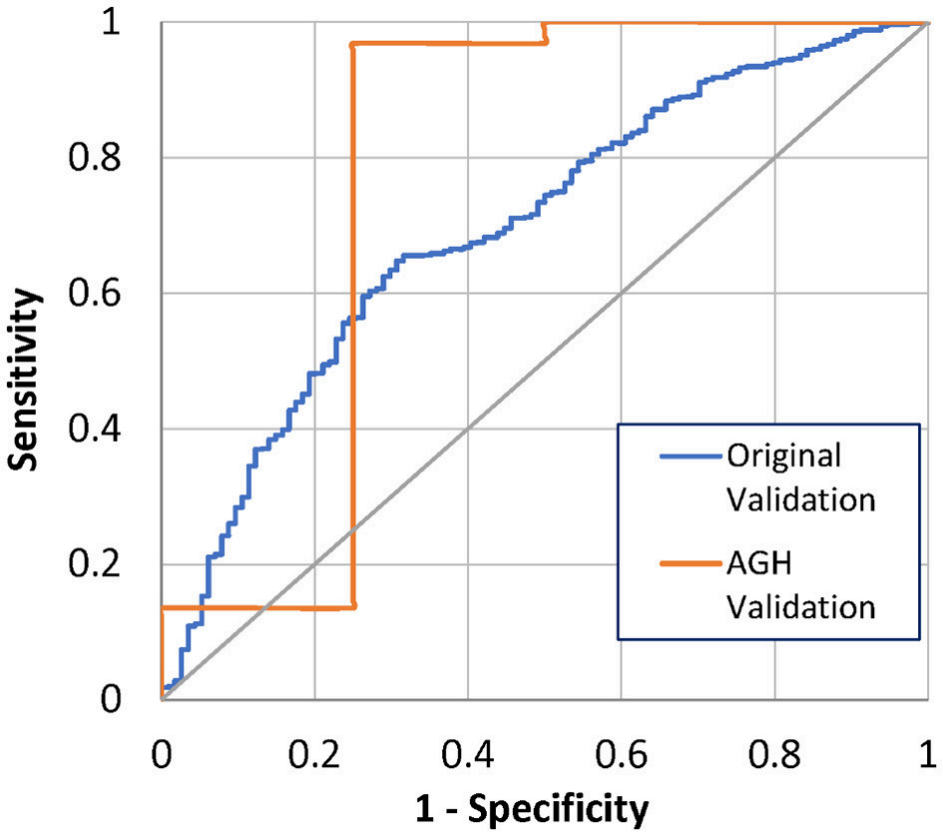
ROC curves for 1-month mortality from original and AGH-specific validation.

One month after implant, 4 (4%) of the 100 AGH patients had died. The 1-month mortality model correctly predicted 3 out of the 4 deaths (75%) and predicted 87 out of 96 alive patients (91%), using a threshold of 50% (Table 2). The ROC AUC was 78%, with a 95% confidence interval (CI) of 0.36–1.0. This is performance is comparable to the original model validation of 70% ROC AUC, with CI 0.65–0.74 (Figure 1). Comparison of the ROC AUCs with DeLong's test yielded *p*-value = 0.71, no statistical difference in performance.

**Table 2:** 1-Month mortality model performance.

	**Mortality at 1 month**	**Survival at 1 month**	**Total**
Actual outcome	4	96	100
Predicted^*^	3	87	90
Performance	75% Sensitivity (95% CI 0.22–0.99)	91% Specificity (95% CI 0.82–0.95)	90% Accuracy

* *Based on predictive survival above 50%*.

**Figure 2:**
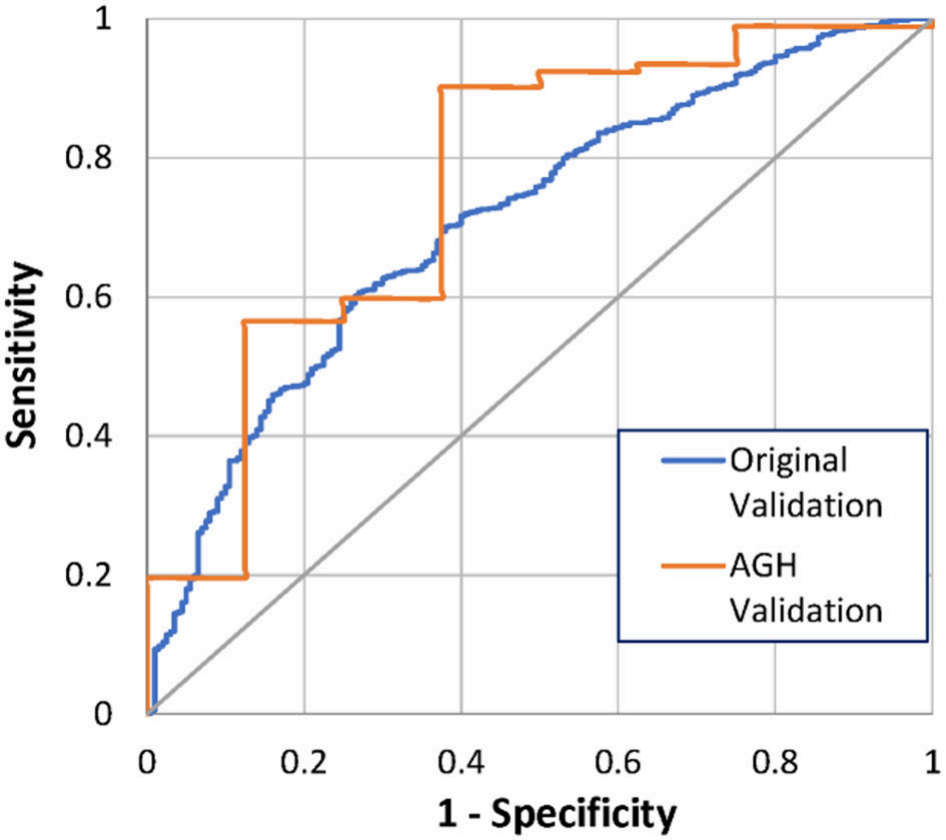
ROC curves for 3-months mortality from original and AGH-specific validation.

At 3 months after implant, 8 (8%) of the 100 patients had died. The Bayesian mortality model correctly predicted 4 of the 8 deaths (50%) and 83 of the 92 living patients (90%), using a mortality risk threshold of 50% (Table 3). The ROC AUC for the model performance was 76% with 95% CI 0.56–0.96. This is comparable to the original model test validation of 71%, with 95% CI 0.67–0.75 (Figure 2). Comparison of the ROC AUCs with DeLong's test yielded *p*-value = 0.61, no statistical difference in performance.

**Table 3:** 3-Months mortality model performance.

	**Mortality at 3 months**	**Survival at 3 months**	**Total**
Actual outcome	8	92	100
Predicted^*^	4	83	87
Performance	50% Sensitivity (95% CI 0.17–0.83)	90% Specificity (95% CI 0.82–0.95)	87% Accuracy

* *Based on predictive survival above 50%*.

**Figure 3:**
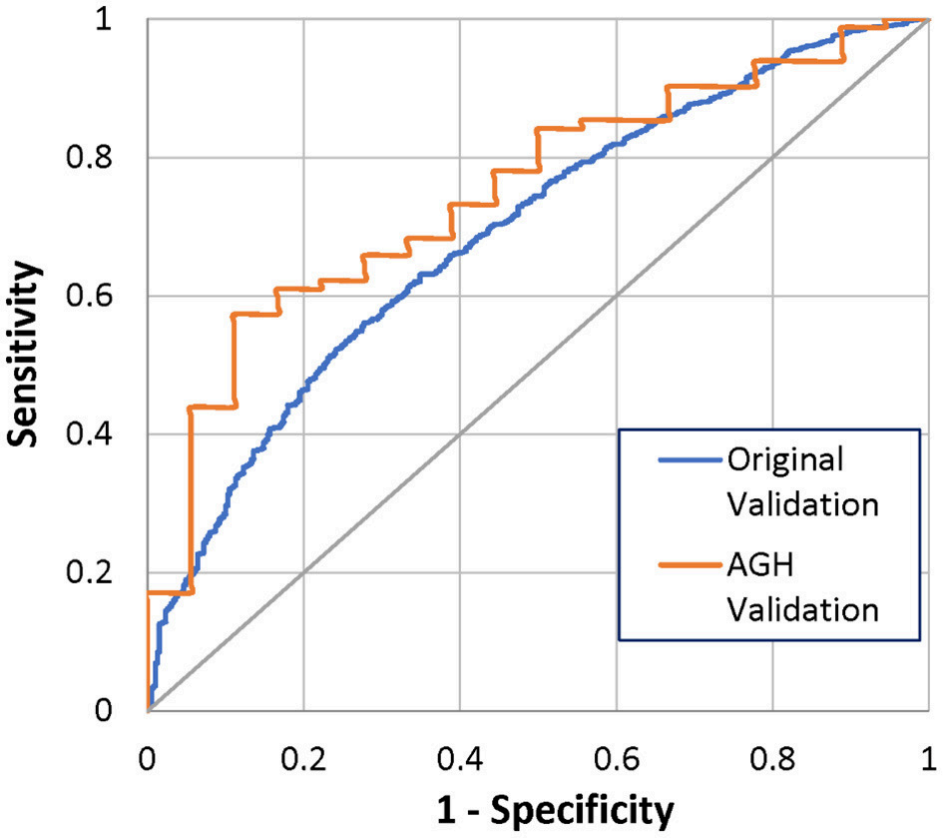
ROC curves for 12-months mortality from original and AGH-specific validation.

By 12 months after implant, 18 (18%) of the 100 patients had died. The Bayesian mortality model correctly predicted 6 of the 18 deaths (33%) and 73 of the 82 living patients (89%), using a mortality risk threshold of 50% (Table 4). The ROC AUC for the model performance was 75%, 95% CI 0.65–0.87, which was comparable to the original model validation of 69%, 95% CI 0.66–0.72 (Figure 3). Comparison of the ROC AUCs with DeLong's test yielded *p*-value = 0.28, no statistical difference in performance.

**Table 4:** 12-Months mortality model performance.

	**Mortality at 12 months**	**Survival at 12 months**	**Total**
Actual outcome	18	82	100
Predicted^*^	6	73	79
Performance	33% Sensitivity (95% CI 0.14–0.59)	75% Specificity (95% CI 0.80–0.94)	79% Accuracy

* *Based on predictive survival above 50%*.

## Discussion

The Bayesian models for mortality derived on INTERMACS data performed with ROC AUCs of 78%, 76%, and 75% in a single center retrospective cohort for 1, 3, and 12 months post-LVAD implant, respectively. We had previously reported ROC AUCs of 70, 71, and 69% with a validation cohort from INTERMACS. All three mortality models performed comparably in the AGH patient dataset, indicating that these models have utility for prospective patient validation at this LVAD implant center.

Verifying model performance on a center's specific patient population is especially important given the influence of institutional experience on outcomes. This has been illustrated by the Heartmate II Risk Score, which includes institution implant volume as a statistically significant predictor for mortality outcomes (10). Additionally, an assessment of implant center volume on 1-year mortality of destination therapy (DT) patients found that low volume centers had a higher mortality rate (11). Similar relationships have been reported for transplant graft survival (12) and right heart failure-associated mortality (13). Since AGH is an experienced, high volume implant center, the models may perform better there than in a lower implant volume institution.

In addition to different in hospital experience, the mix of patient health status and strategy of patient management may impact model performance. There were significantly more patients with severe heart failure, as indicated by the percentage of patients with INTERMACS 1 and 2 profiles, at AGH. However, the mortality rates for AGH patients at each time point were comparable to the mortality rates in the INTERMACS population. Subjectivity in patient classification (14) or experience in patient management may contribute to the rate of patient survival. AGH also had significantly more patients who were BTT and fewer who were DT, compared to the INTERMACs population. However, this distribution of patients is in line with the INTERMACs cohort, where DT patients are more often INTERMACs profile 3 and 4 (15).

Despite the data cleaning step at AGH, there were 1,184 fields that were not recorded. A strength of using the Bayesian modeling for this risk tool is that it is robust to missing information when making predictions, as demonstrated by the resulting ROC AUCs. Whether having no missing data would improve the model performance remains unknown. However, it is unlikely that any institution can have a value for every possible patient variable, especially in cases where rapid patient deterioration requires an emergent decision. The use of Bayesian methods makes these models attractive for real world use.

**Figure 4:**
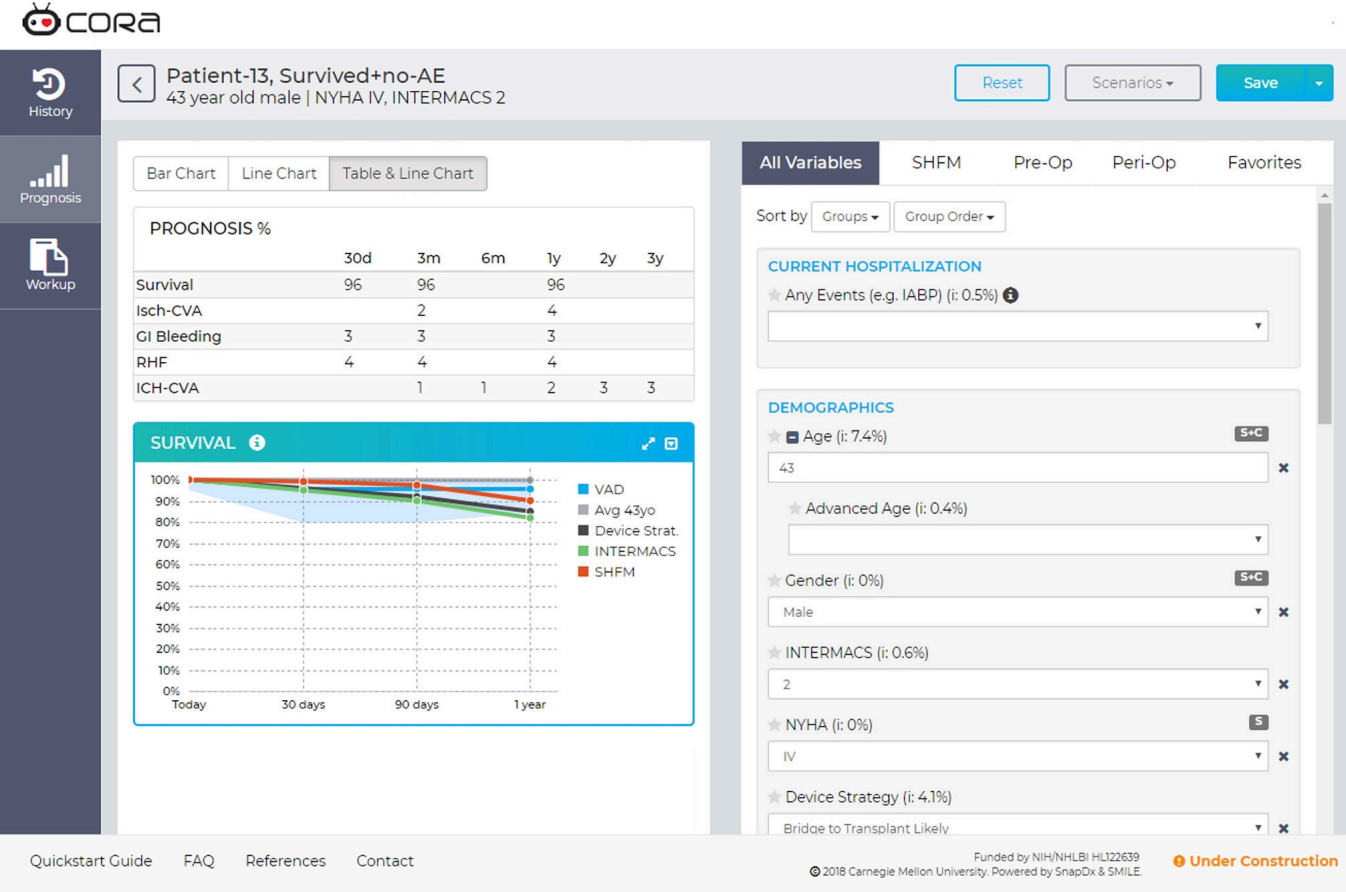
Screen capture of the CORA decision support tool. The myCORA app shows risk predictions for survival, ischemic stroke (Isch-CVA), recurrent GI bleeding, right heart failure (RHF), and hemorrhagic stroke (ICH-CVA). Data are presented in the Prognosis table as percent probability at different time points. In the Survival line graph, the predicted survival for the patient on an LVAD is shown in the blue “VAD” line. The gray “Avg 43” presents the survival of a non-sick 43-years-old, derived from census data. The dark gray line “Device Strat” presents the survival prediction for all patients with the same device strategy (e.g., Bridge to Transplant). The green line “INTERMACS” presents the survival for all patients with the same INTERMACS Profile (e.g., profile 3). Finally, the orange line “SHFM” is the survival prediction for the patient calculated with the Seattle Heart Failure Model.

The models assessed in this analysis are available at app.myCORA.org with an institutional login, as part of the Cardiac Outcomes Risk Assessment (CORA) decision support tool for physicians (Figure 4). This tool has begun to be prospectively evaluated by the multidisciplinary team at the weekly transplant meetings at AGH to assess its performance and impact on clinician decision making. At present, patient data will be entered manually into the tool by a VAD coordinator, but work is in progress to allow for integration with the electronic health record system. Predictive models for post-LVAD adverse events are being developed to add to the CORA tool (e.g., ischemic stroke, recurrent gastrointestinal bleeding, and right heart failure) and will be evaluated for performance with the same single center, retrospective validation methodology.

## Conclusion

By validating the model set at a single clinical site, performance can be demonstrated for the patient population served at that particular site and for the unique surgical and medical management style of the clinicians. This exercise is imperative to confirm the utility of the mortality models for clinical decision making. Future work will be to prospectively test the model performance in the AGH multidisciplinary team meeting setting, to evaluate utility in real life decision making.

## Author contributions

LL conceptualized the study, analyzed the data for this research, and wrote the manuscript. MK facilitated the data collection and provided input on study design, as well as provided substantial edits to the manuscript. SB edited the manuscript and provided insight for additional discussion. SM and JA provided input on study design, provided insight for discussion, and edited the manuscript.

### Conflict of interest statement

The authors declare that the research was conducted in the absence of any commercial or financial relationships that could be construed as a potential conflict of interest.
