# Supplementary figures and images for: Retrospective Evaluation of Bayesian Risk Models of LVAD Mortality at a Single Implant Center
Source: Front Med (Lausanne). 2018 Oct 2;5:277. doi: 10.3389/fmed.2018.00277 (PMC6176112; doi:10.3389/fmed.2018.00277)

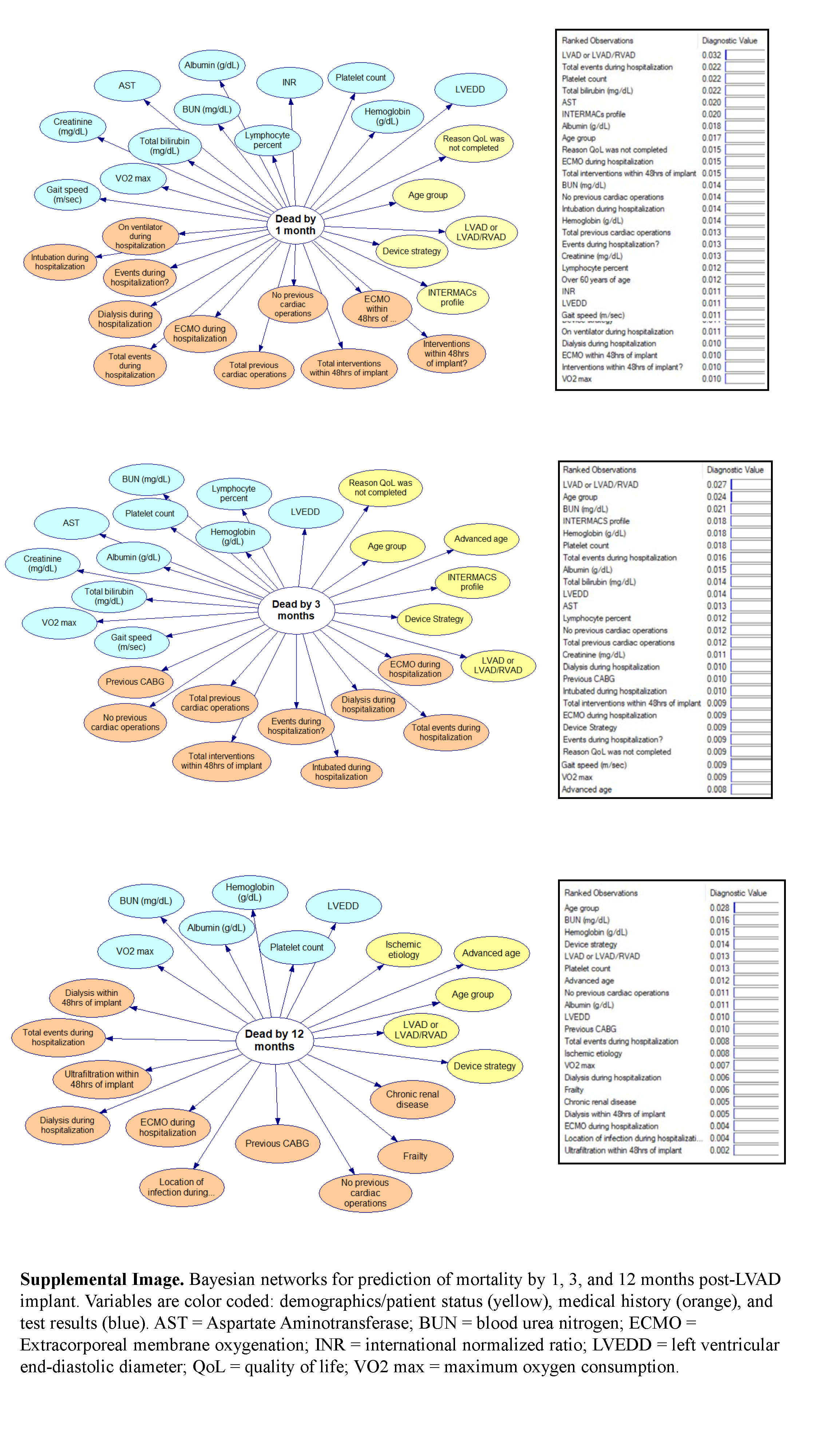

Supplement: Supplementary file 1 [file Image_1.tif]
